# Phytonutrients of Bitter Apricot Seeds Modulate Human Lipid Profile and LDL Subfractions in Adults with Elevated Cholesterol Levels

**DOI:** 10.3390/ijerph19020857

**Published:** 2022-01-13

**Authors:** Jana Kopčeková, Anna Kolesárová, Marianna Schwarzová, Anton Kováčik, Jana Mrázová, Martina Gažarová, Petra Lenártová, Peter Chlebo, Adriana Kolesárová

**Affiliations:** 1 Institute of Nutrition and Genomics, Faculty of Agrobiology and Food Resources, Slovak University of Agriculture in Nitra, 949 76 Nitra, Slovakia; marianna.schwarzova@uniag.sk (M.S.); jana.mrazova@uniag.sk (J.M.); martina.gazarova@uniag.sk (M.G.); petra.lenartova@uniag.sk (P.L.); peter.chlebo@uniag.sk (P.C.); 2 Institute of Food Sciences, Faculty of Biotechnology and Food Sciences, Slovak University of Agriculture in Nitra, 949 76 Nitra, Slovakia; anna.kolesarova@uniag.sk; 3 Institute of Applied Biology, Faculty of Biotechnology and Food Sciences, Slovak University of Agriculture in Nitra, 949 76 Nitra, Slovakia; anton.kovacik@uniag.sk (A.K.); adriana.kolesarova@uniag.sk (A.K.)

**Keywords:** apricot seeds, phytonutrients, lipid profile, cholesterol, LDL subfractions

## Abstract

The objective of the present study was to evaluate the effect of short-term consumption of bitter apricot seeds phytonutrients on cardiovascular risk factors with a special focus on LDL cholesterol subfractions using the Lipoprint system. A group of 34 adult volunteers (21 female/13 male) consumed 60 mg kg^−1^ of body weight of bitter apricot seeds daily for 42 days. Subjects were divided into two groups: one with normal cholesterol levels (NTC) and one with elevated total cholesterol levels (ETC). Blood serum levels of total cholesterol (T-C), low-density cholesterol (LDL-C), high-density cholesterol (HDL-C), and triglycerides (TG) did not change significantly (*p* > 0.05) in NTC group. However, there were significant decreasing of T-C (*p* ˂ 0.05) and LDL-C (*p* < 0.01) in ETC group. The LDL_1_, LDL_2_, and atherogenic LDL_3−7_ subfractions progressively decreased after 42 days of apricot seeds consumption in ETC group (*p* < 0.05). Apricot seeds consumption was associated with a significant increase in the mean LDL particle size especially in ETC group (*p* ˂ 0.01). The results of the present study support the hypothesis that daily consumption of bitter apricot seeds for 42 days positively modified the lipoprotein profile in the group with elevated total cholesterol.

## 1. Introduction

Cardiovascular disease (CVD) remains the world’s leading cause of morbidity and mortality [1,2]. Atherosclerosis is the dominant cause of cardiovascular disease [3], including myocardial infarction (MI), heart failure, stroke, and claudication [4]. Atherosclerosis is a multifactorial disease that is thought to be primarily inflammatory in origin [5]. The pathogenesis of atherosclerosis involves a complex interplay of endothelial dysfunction, inflammation, lipid accumulation, vascular smooth muscle cell proliferation, matrix transformation and calcification, and persistent inflammation and ultimately leads to the formation of atherosclerotic plaque [2,6,7,8].

A major risk factor for CVD is dyslipidemia, which occurs mainly in adults worldwide and is defined as an abnormal blood lipids level [9]. Dyslipidemia is characterized by elevated levels of low-density lipoproteins (LDL-C), small and very low-density lipoproteins (VLDL), and intermediate-density lipoproteins (IDL), triglyceride (TG), and low serum levels of high-density lipoprotein cholesterol (HDL-C) and its lipoprotein subfractions, which correlate with an increased risk for CVD [10,11,12,13]. Hypercholesterolemia was reported as the highest attributable risk factor for atherosclerosis and subsequent coronary heart disease (CHD) in a given population. Although increased LDL-C is considered a major risk factor for cardiovascular disease [14,15], a number of cardiac events occur in people without clinically abnormal LDL-C concentrations [16]. The atherogenic potential of LDL particles is not just associated to their concentration but also to their heterogeneity with regard to lipid composition, particle size, and density [17].

In recent decades, lipoprotein research has focused on the characterization of atherogenic and non-atherogenic lipoprotein profiles [18]. This may be due to the fact that lipoprotein particles consist of a heterogeneous group of subfractions differing not only in size and density but also in chemical composition and physiological function [19,20,21]. In the presence of the seven fractions of LDL cholesterol, which important for the distribution on anti-atherogenic fractions LDL_1–2_ and atherogenic small dense lipoproteins LDL_3–7_ (sdLDL), their role in the pathogenesis of coronary atherosclerosis was confirmed [22,23]. Studies have demonstrated that the concentration of sdLDL was positively correlated with the incidence of CVD [24]. Smaller and denser LDL particles are considered as an atherogenic risk factor for CVD due to their greater tendency to oxidize and their permeability through the endothelium of arterial walls [22,25]. Large LDL particles (diameter ≥ 25 nm) are considered a non-atherogenic phenotype (pattern A) and small, dense LDL particles with sizes 19.0–20.5 nm are considered an atherogenic phenotype (pattern B) [26,27]. Monitoring and control of LDL-C and HDL-C levels is considered necessary to reduce the risk of cardiovascular disease [28].

The diet is an important determinant of serum cholesterol, but dietary cholesterol has only a modest contribution to plasma concentrations of LDL-C [29]. Diet can affect CVD directly by modulating the composition of vascular plaques and indirectly by affecting the rate of aging [30]. Previous epidemiological studies indicate that high consumption of foods rich in bioactive compounds has a positive effect on human health and could diminish the risk of numerous diseases, such as cancer, heart disease, stroke, Alzheimer’s, diabetes, cataracts, and age-related functional decadence [31,32]. Phytonutrients or phytochemicals are natural bioactive compounds obtained from plants that perform specific biological activities and modify different physiological functions to improve general human health [33,34]. They have specific pharmacological effects that may affect aging, allergies, antioxidant defense, blood pressure, bones, cancer, the central nervous system, diabetes, the gastrointestinal tract, immunity, inflammation, lipidemia, the liver, microbes, pain, and much more [35].

Bitter apricot seeds are important source of phytochemicals with strong chemo-preventive activity [36,37]. They contain phenolic compounds, terpenoids, oil, and bioactive proteins and peptides [37] and exhibited higher antioxidative activity than flesh of the fruit [38]. The use of bitter apricot seeds in human nutrition is limited compared to sweet seeds due to the content of the toxic, cyanogenic glycoside amygdalin [39]. Various pharmacological studies demonstrated anti-inflammatory [40], anti-asthmatic [41,42], analgesic, anti-mutagenic [36], anti-tussive [43], antioxidant [36,44,45,46], anti-cancer [44,47,48,49,50,51,52,53,54,55,56], and anti-microbial [36,45] effects of apricot seeds. Our previous reports described the effects of bitter apricot seeds consumption on body composition, blood serum lipids, and other risk factors for CVD [57] in healthy volunteers. This study is the first trial to evaluate the effects of short-term consumption of bitter apricot seeds phytonutrients on cardiovascular risk factors, with a special focus on LDL cholesterol subfractions of adults with elevated cholesterol levels.

## 2. Materials and Methods

### 2.1. Participants and Study Design

A group of 34 adult volunteers (21 female/13 male; mean age 38.91 ± 10.77 years) participated in a 6-week interventional program. Subjects were divided into two groups: those with normal cholesterol levels, i.e., NTC (total cholesterol < 5.0 mmol/L, 7 male and 14 female; average age 36.05 ± 7.85), and those with elevated total cholesterol levels, i.e., ETC (total cholesterol ≥ 5.0 mmol/L, 6 male and 7 female; average age 43.54 ± 13.38) in order to evaluate the individual influence of bitter apricot seeds on the blood lipid profile.

The volunteers had to meet the following inclusion criteria: willingness to participate in a 6-week interventional program, apparently healthy women and men, age 20–60 years, stable body weight (±3 kg) during the last 3 months, and alcohol consumption ≤ 30 g/day. Exclusion criteria included an inability to give informed consent; use of cholesterol lowering medications or supplements; chronic diseases (i.e., cardiovascular diseases, diabetes, inflammatory diseases, cancer, allergy); thyroid abnormalities; active liver disease; use of hypolipidemics, corticosteroids, etc.; pregnancy or plans to become pregnant during the study and breastfeeding; tobacco, alcohol, or drug addiction; no intake of any nutritional supplements (vitamins, minerals, antioxidants, and flavonoids); or parallel participation in other dietary intervention study.

A written informed consent was obtained from all the participants prior to their involvement in the study. They were informed of all risks, discomforts, and benefits. This study was conducted according to the guidelines of the Declaration of Helsinki and approved by the Ethics Committee at the Specialized Hospital St. Zoerardus Zobor, n. o. Nitra, Slovak Republic (protocol number 030809/2015).

### 2.2. Dietary Intervention

Volunteers consumed 60 mg/kg of body weight of bitter apricot seeds divided into several daily doses for 42 days period. The subjects were instructed not to change the composition of their usual diet and their nutrition pattern, to consume approximately one seed each hour (by chewing as thoroughly as possible), and drink water after each consumption.

Apricot seeds were supplied by company TRASCO, Ziar nad Hronom, Slovak Republic. Contents of nutrients were determined by the following methods of AOAC [58]: ash (residue after ignition at 535 °C), crude protein (N determined by Kjeldahl method ȕ 6.25) using Kjeltec Auto 1030 (Foss, Denmark), fat content by Soxhlet method for fat extraction using Tecator Soxtec System HT6 (Foss, Denmark), and crude fiber by Henneberg-Stohmann method and total sugars by Luff-Schoorl titration. The content of fatty acids as a percentage in crude fat was determined using Agilent 6890 A GC (Agilent Technologies, Wilmington, DE, USA). Mineral content in apricot seeds was determined by atomic absorption spectroscopy using AVANTA analyzer (GBC Scientific Equipment, Braeside, Australia). Thin Layer Chromatography (TLC) was performed for the analysis of amygdalin content in bitter apricot seeds. Polyphenol compounds were determined by Agilent 1260 HPLC-DAD (Agilent Technologies, Wilmington, DE, USA). Composition of apricot seed used in this study is presented in Table 1.

We used the 24-h dietary recall, where respondents provides information about the type and quantity of all food and beverages consumed during the previous 24-h period. We evaluated the data using Mountberry nutritional and fitness software (2011, version 1.1) (Wellberry, s.r.o., Nitra, Slovak Republic). This software is designed for a complete analysis of food, meals, and recipes based on the composition of the raw ingredients.

Anthropometric parameters, including body weight (BW), waist circumference (WC), waist-hip ratio (WHR), body fat mass (BFM), visceral fat area (VFA), body mass index (BMI); traditional lipid profile, including total cholesterol (T-C), low-density cholesterol (LDL-C), high-density cholesterol (HDL-C), triglycerides (TG), and lipoprotein subfractions (VLDL, IDL-A, IDL-B, IDL-C, LDL_1_, LDL_2_, LDL_3–7_); liver enzymes, including alanine aminotransferase (ALT), aspartate aminotransferase (AST), alkaline phosphatase (ALP), and gamma glutamyl transferase (GGT); and inflammatory response markers, such as high-sensitivity C-reactive protein (hs-CRP) and creatine kinase (CK), were monitored at baseline of apricot seeds consumption (day 0) and after 6 weeks of consumption (day 42).

### 2.3. Anthropometric Data

At baseline as well as 6 weeks after of apricot seeds consumption, volunteers were assessed for weight and height using standard procedures. Body height was measured on the outpatient electronical medical scales Tanita WB-300 in the upright standing position, without shoes. Body weight, waist circumference, hip circumference, and body composition (BFM, %BFM, and VFA) were diagnosed by multi-frequency bioelectrical impedance analysis (MFBIA) by InBody 720 (Biospace Co., Seoul, Korea). The body mass index (BMI) was calculated by dividing the body weight in kilograms by the square of the height in meters. WHR was calculated by dividing the waist measurement by the hip measurement [59].

### 2.4. Blood Sample Collection and Clinical Analysis

Blood samples were obtained at baseline (day 0) and after 6 weeks after (day 42) of apricot seeds consumption. Venous blood from brachial vein of one arm was collected in the morning after 8 h of fasting in a standard manner using a vacutainer tube containing ethylendiaminetetra-acetic acid (EDTA) (2.7 mL) and serum gel (7.5 mL) by a qualified phlebotomist. Whole blood was centrifuged at 3000× *g* for 10 min at 4 °C. Plasma and serum were separated and stored at −80 °C until the analyses.

The fasting serum T-C and TG, liver enzymes (ALT, AST, ALP, and GGT), and CK were measured using commercial kits DiaSys (Diagnostic Systems GmbH, Holzheim, Germany) on the Randox RX Monza analyzer (Randox Laboratories Ltd., Crumlin, United Kingdom). HDL-C and hs-CRP were measured by automatic biochemical analyzer Biolis 24i Premium (Tokyo Boeki Machinery Ltd., Tokyo, Japan) using the commercial kit Randox for HDL-C and Diasys for hs-CRP. The LDL-C level was calculated using the Friedewald equation as T-C-HDL-C-(TG/2.2) in mmol/L [60].

The LDL subfractions and mean LDL particle size were determined from plasma using the analyzer Lipoprint^®^ with commercial “Lipoprint LDL Kit” (Quantimetrix Corp., Redondo Beach, CA, USA) according to the procedure provided by the manufacturer. This method uses linear electrophoresis on a nondenaturing polyacrylamide gel to separate and quantify lipoprotein subfractions. This method allows to separate the LDL subfractions (1 and 2 are large LDL, and 3–7 subfractions are small dense LDL), very low-density lipoprotein (VLDL) fractions, as well as the intermediate-density lipoprotein (IDL) C, B, and A.

Lipoprint reports LDL phenotype based on particle size as non-atherogenic phenotype A (≥26.8 nm), intermediate phenotype AB (26.51–26.79 nm), or atherogenic phenotype B (≤26.5 nm).

### 2.5. Statistical Analysis

Statistical analysis was carried out using the Statistica Cz version 10 (TIBCO Software, Inc., Palo Alto, CA, USA) and MS Excel 2007 (Microsoft Corporation, Redmond, WA, USA). Shapiro–Wilk test was used for testing normality. All the data were expressed as the mean ± standard deviation (SD). We used a paired *t*-test for normally distributed variables and Wilcoxon Signed-Ranks Test for not normally distributed variables. Analysis of variables was performed by one-way analysis of variance (ANOVA). ANCOVA test was used to assess if the differences seen in ANOVA persisted after adjustment for body mass index (BMI) and sex. The statistical significance was established at *p* ˂ 0.05. For all outcomes, within-group Cohen’s *d* effect sizes were determined by calculating the mean difference between two groups and then dividing the result by the pooled standard deviation.

## 3. Results

A group of 34 adult volunteers ranging in age from 23 to 65 years with a mean age of 38.91 ± 10.77 years participated in the present study. Subjects were divided into the following two groups: those with normal cholesterol levels, i.e., NTC (total cholesterol < 5.0 mmol/L, 7 male and 14 female), and those with elevated total cholesterol levels, i.e., ETC (total cholesterol ≥ 5.0 mmol/L, 6 male and 7 female). Characteristics of the study subjects are summarized in Table 2.

### 3.1. Effect of Bitter Apricot Seeds Consumption on Anthropometric Characteristics

The results of anthropometric characteristics (BW, BFM, VFA, and BMI) of the volunteers after six weeks of apricot seeds consumption are shown in Table 3. In the NTC group, we recorded a significant decrease of BW, BFM, and BMI and, on the contrary, in the ETC group, a significant increase in BW, VFA, and BMI (*p* ˂ 0.05) after 42 days of consumption of apricot seeds. The Cohen’s *d* effect sizes of anthropometric characteristics for the change from baseline to week 6 was ˂0.1 for both groups.

### 3.2. Effect of Bitter Apricot Seeds Consumption on Traditional Blood Serum Lipid Profile and Fasting Glucose

Lipid profile of subjects at baseline, and at the end of the intervention periods are shown in Table 4. Blood serum levels of T-C, LDL-C, HDL-C, and TG did not change significantly (*p* > 0.05) after 42 days of bitter apricot seeds consumption in NTC group (Cohen’s *d* ˂ 0.1). However, there was a significant decrease of T-C (*p* ˂ 0.05) and LDL-cholesterol (*p* < 0.01) in ETC group after 42 days of apricot seeds consumption. The Cohen’s *d* effect sizes of LDL-C for the change from baseline to week six in NTC group was ˂0.1 and in ETC was 0.701.

### 3.3. Effect of Bitter Apricot Seeds Consumption on Lipoprotein Subfractions

Table 5 presents changes in the distribution of cholesterol in different lipoprotein subfractions and mean LDL particle size in response to the intervention. After 42 days of consumption of apricot seeds, there were significant differences in the IDL-B (*p* ˂ 0.01) and LDL_2_ (*p* ˂ 0.05) in NTC group. The LDL_1_ and LDL_2_ subfractions progressively decreased during the intervention (*p* < 0.01) in ETC group, but there were always significant differences in the atherogenic LDL_3–7_ (*p* ˂ 0.05). Furthermore, apricot seeds consumption was associated with a significant increase in the mean LDL particle size, especially in ETC group (*p* ˂ 0.01). Cohen’s *d* effect sizes for the change from baseline to week six was small for all lipoprotein subfraction (0.070–0.453) in NTC group. In the ETC group, Cohen’s *d* effect sizes were small for VLDL, IDL-A, IDL-B, and IDL-C; medium for LDL_1_ (0.715) and total LDL (0.626); and large for LDL_2_ (1.220), LDL_3–7_ (0.819), and mean LDL particle (−1.086).

**Figure 1:**
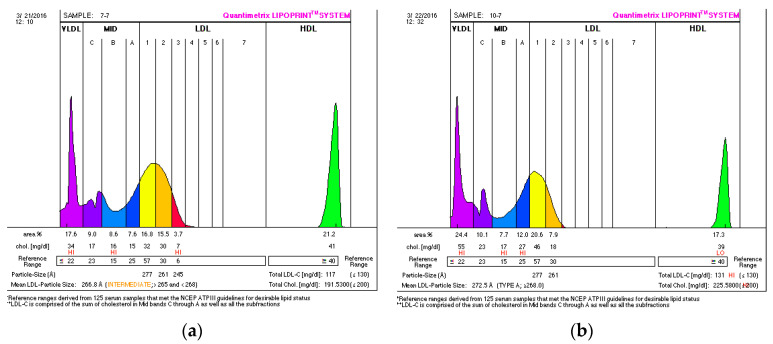
A representative lipoprotein profile of one proband from NTC group at baseline (**a**) and after consumption (**b**) of bitter apricot seeds. Atherogenic subfractions LDL_3–7_ are in red; the large—less atherogenic subfractions LDL_1_ and LDL_2_ are in yellow.

In this study, we found some atherogenic LDL subfractions (LDL_3–7_) in three volunteers from the NTC and five volunteers from the ETC group. At the end of the study, we found a manifestation of atherogenic subtractions only in one man from the NTC group and in one man from the ETC group. After the six-week bitter apricot seeds consumption, the number of subjects with fractions LDL_3–7_ decreased in both group (by 9.53% in NTC group and by 30.77% in ETC group). We found intermediate-pattern AB only in two volunteers in the NTC group, but after a six-week intervention, only one participant was classified as intermediate-pattern (AB), while all others were evaluated as a non-atherogenic lipoprotein profile type (A). Figure 1 shows a representative lipoprotein profile of proband from NTC group in response to the intervention.

### 3.4. Effect of Bitter Apricot Seeds Consumption on High-Sensitivity CRP, CK, and Liver Enzymes

High-sensitivity CRP, CK, and the enzyme activities of AST, ALT, ALP, and GGT during consumption of apricot seeds are shown in Table 6. After the intervention, non-significant decrease of hs-CRP and non-significant increase of CK was recorded in both groups (*p* > 0.05). Serum AST activity decreased significantly (*p* < 0.01), and activity of GGT increased significantly (*p* < 0.05) in NTC group. At the start of the study, three participants in this group had GGT values lower than their physiological values, but after 42 days, they had GGT in the normal range. The activities of liver enzymes did not change significantly (*p* > 0.05) after 42 days of bitter apricot seeds consumption in ETC group. In this study, the average values of the activity of liver enzymes in all the participants were in the range of physiological values. The Cohen’s *d* effect sizes of high-sensitivity CRP, CK, and liver enzymes for the change from baseline to week six was ˂0.1 for both groups.

### 3.5. Comparison of Monitored Parameters after Nutrition Intervention Adjusted for Body Mass Index (BMI) and Sex

The covariance analysis (ANCOVA) showed that the differences observed in the monitored variables were not affected by body mass index and only to a small extent by sex, which may also be related to the gender imbalance of the NTC group, the predominance of women compared to men (66.7% vs. 33.3%), and the possible impact of age. The mean age in this group was 37.50 ± 9.13 years for women and 33.14 ± 3.18 years for men. There were no significant interactions for changes in all parameters adjusted for BMI in ETC group. The effect of sex in the ETC group was manifested only in the HDL-C, which was expected, as women have physiologically higher HDL compared to men (Table 7).

## 4. Discussion

The aim of this study was to evaluate the effects of short-term consumption of bitter apricot seeds phytonutrients on cardiovascular risk factors with a special focus on LDL cholesterol subfractions of adults with elevated cholesterol levels.

Dietary recommendations for the prevention of chronic diseases have now shifted towards diets high in plant foods and low in animal foods [61,62,63] not only because of low saturated fat and cholesterol but also because of the significant amounts of micronutrients and bioactive compounds [64,65]. This dietary pattern has been associated with lower risk of CVD [66,67,68,69] and is widely recommended for heart health [70].

In recent years, seeds and nuts have received growing attention due to high nutraceutical and therapeutic value of their bioactive components [71,72,73,74]. Some of the reported health benefits derived from nut and seed consumption are control of body weight and blood pressure, reduction of coronary heart disease, and reduction of levels of blood cholesterol and triacylglycerols [75,76]. There is substantial evidence that increased consumption of seeds is associated with lower risk of CVD and T2DM or a significant reduction in CVD risk factors, such as serum cholesterol or blood pressure [77]. In addition, nuts and edible seeds provide antioxidant, anti-microbial, anti-inflammatory, anti-mutagenic, anti-cancer, anti-diabetic, and glucoregulatory properties [75,78]. Apricot seeds and almond oil are rich in mono- and polyunsaturated fatty acids [79]. A meta-analysis of clinical trials concluded that nut intake led to a significant beneficial effect on triglycerides, total cholesterol, LDL, and Apo B [80]. In an earlier study, almonds in the daily diet reduced LDL-C by as much as 9.4%, reduced the LDL:HDL ratio by 12.0%, and increased HDL-C by 4.6% [81]. Zibaeenezhad et al. [82] found that supplementation of *Amygdalus scoparia* kernel oil had a positive effect on lowering serum triglycerides in patients with dyslipidemia without a significant effect on serum cholesterol levels. In our experiment, we observed a positive effect on both triglycerides and total cholesterol levels in the ETC group.

Our and previous studies indicate a high concentration of important bioactive substances with potential health benefits. The main bioactive components of apricot oil are fatty acids, tocopherols, and phenolic compounds [83,84,85]. Apricot kernel oil contains some biologically active substances, such as β-carotene (61.05 mg/g), tocopherols (50.76 mg/100 g), phenolic compounds, campesterol (11.8 mg/100 g), stigmasterol (9.8 mg/100 g), sitosterol (177.0 mg/100 g), and provitamin A [86,87]. Seed oils from Prunus species contain high amounts of recommended monounsaturated oleic acid (60–70.9%), moderate content of linoleic acid (20–30%), and low amounts of saturated fatty acids [86,87,88], which is also in line with our results. Polyunsaturated and monosaturated fatty acids are important for normal growth and development and are suggested to play an important role in modulation of cardiovascular inflammatory diseases and cancer [89]. Amygdalin, a bioactive component of bitter apricot seeds, has controversial functions in cancer therapy [90], Jiagang et al. [91] first examined its therapeutic effect in atherosclerosis. They found decreased TG and T-C levels (*p* < 0.05) in mice treated by amygdalin (1 mg/kg). Meanwhile, amygdalin treatment also decreased the LDL-C levels (*p* < 0.05). However, the treatment of amygdalin did not induce any decline in HDL-C levels (*p* > 0.05). Of the phenolic compounds in tested bitter apricot seeds, we mainly determined gallic acid (30.1 mg/g) and rutin (11.3 mg/g). Gallic acid (GA) is one of the most important herbal products that has beneficial effects on CVD [92,93,94]. A small amount of GA (in the range of daily consumption in central Europe) prevents oxidative DNA damage and reduces markers that reflect inflammation and increased risks of cancer and CVD [95]. Flavonoids, of which rutin is a typical representative, serve a positive role in the treatment of cardiovascular and cerebrovascular diseases [96]. Rutin has a variety of pharmacological actions, including radical reactivity and protective activity against lipid peroxidation, viruses, and acute pancreatitis; thus, it may be used as a treatment for many diseases. Rutin significantly reduced the levels of total cholesterol and LDL-C and also markedly decreased liver enzymes and weight in animals with a high-cholesterol diet [97].

Despite their relatively high energy density, intake of nuts and seeds was actually associated with less weight gain, lower risk of obesity, and lower risk of moderate weight gain in prospective studies [98,99,100]. When comparing changes in body composition, we found a significant decrease in BW, BFM, and BMI in the NTC group, but we observed a significant increase in BW, VFA, and BMI in the ETC group. Hypercholesterolemia is the main risk factor of cardiovascular diseases, such as atherosclerosis, myocardial infarction, stroke, and cerebrovascular diseases. Elevated LDL-C and decreased HDL-C are important risk factors for cardiovascular diseases, particularly for coronary artery disease [101,102]. Despite the causal role of LDL cholesterol in development of atherosclerosis, previous studies have indicated that the association of elevated total cholesterol with myocardial infarction and ischemic heart disease varies greatly with age, with the association being much stronger in younger than older individuals [103,104,105,106]. Age is an uncontrollable risk factor for elevated cholesterol level, which is consistent with our results; NTC subjects were significantly younger in comparison to ETC subjects.

In our previous studies, consumption of bitter apricot seeds also caused significant reductions (*p* < 0.05) in T-C and LDL-C levels in healthy volunteers [57] and also in women of reproductive age [107]. In the present study, the blood serum levels of T-C, LDL-C, HDL-C, and TG did not change significantly (*p* > 0.05) after 42 days of bitter apricot seeds consumption in NTC group; however, there were significant reduction in the total cholesterol (*p* ˂ 0.05) and LDL-cholesterol (*p* < 0.01) in ETC group after 42 days of apricot seeds consumption. A 5% reduction in LDL-cholesterol is significant, as it could reduce risk of CHD from 5% to 15%; every 1% reduction in LDL-C is associated with a decreased risk for CHD of 1% to 3% [108,109,110,111]. A high concentration of plasma triglycerides has been associated with cardiovascular disease risk in humans for several decades [112]. In our study, triglycerides concentrations was not significantly (*p* > 0.05) changed by six weeks of apricot seed consumption in both groups. LDL-C is accepted as a causal risk factor for development of myocardial infarction and atherosclerotic cardiovascular disease [113]. Evidence suggests that lipoprotein particle size and the distribution of cholesterol in LDL and HDL subfractions may be better predictors of CVD than traditional lipid profiles [114,115,116,117]. Although nutraceuticals and herbal medicine have been previously studied as a non-pharmacological management of dyslipidemia [118,119,120,121,122], greater clarity on the effects of these bioactive natural compounds in improving sdLDL for the reduction in the relative risk of CVD is needed. To date, there is no study that has investigated the effect of phytonutrients of bitter apricot seeds on sdLDL levels. There are evidences that the small LDL number was significantly decreased in the pistachio-supplemented diet [37,123], hazelnut-enriched diet [124], and walnuts diet [125]. After 42 days of consumption of apricot seeds, there were significant differences in the IDL-B (*p* ˂ 0.01) and LDL2 (*p* ˂ 0.05) in NTC group. The athero-protective role of LDL_1_ subfraction has been demonstrated [126,127], but the role of LDL_2_ subfraction has not been determined yet [127]. It is assumed that, in the class of LDL lipoproteins, the subfractions LDL_1_ and LDL_2_ are the least atherogenic [23]. In our study, the LDL_1_ and LDL_2_ subfractions progressively decreased during the intervention (*p* < 0.01) in ETC group, but there was always significant decrease in the atherogenic LDL_3–7_ (*p* ˂ 0.05). Zitnanova et al. [126] demonstrated the protective role of IDL-A in the atherogenic process; in this study, IDL-A increase was insignificant (*p* > 0.05) in both groups.

The present work studied adults with normal (NTC) or elevated cholesterol levels (ETC), and some atherogenic LDL subfractions (LDL_3–7_) at the baseline were found in three volunteers from the NTC and five volunteers from the ETC group. According to Oravec et al. [127] the atherogenic lipoprotein profile might be present in about 6% of normolipidemic, young healthy individuals. At the end of the study, we found the LDL_3–7_ fraction only in one man from the NTC group and in one man from the ETC group. Nonetheless, the main conclusion of this study was that after the six-week bitter apricot seeds consumption, the number of subjects with fraction LDL_3–7_ decreased in both group (by 9.53% in NTC group and by 30.77% in ETC group). Furthermore, apricot seeds consumption was associated with a significant increase in the mean LDL particle size, especially in ETC group (*p* ˂ 0.01) with large Cohen’s *d* effect sizes from baseline to week six. It is interesting to note that we found intermediate-pattern AB only in two volunteers in the NTC group, but after a six-week intervention, only one participant was classified as intermediate-pattern (AB), while all others were evaluated as a non-atherogenic lipoprotein profile type (A). Previous studies have shown that particle size represents a threefold increase in cardiovascular risk, independent of other lipid parameters [117,128].

Apricot seeds are used to enrich noodles [129], are added to bakery products as whole seeds or are ground, and are also consumed as an appetizer [130]. Apricot seeds can become part of the normal daily diet, such as nuts, flax seeds, and others, to increase nutritional value, but their consumption is limited because of their content of amygdalin.

This study had certain limitations. The major limitation of our study is the small number of participants, which could decrease the statistical power to detect differences during the follow-up. The next limitation of our study is the age-unbalanced groups of participants; subjects in the group with hypercholesterolemia were older in comparison to subjects with normal levels of total cholesterol. Whole-body cholesterol metabolism is maintained by a highly coordinated balancing act between cholesterol ingestion, synthesis, absorption, and excretion, and ageing interacts with these processes. Another limitation is the short duration of this intervention study. Further studies with a larger sample size, longer duration, and age-balanced participants are needed to further investigate the extent to which apricot seeds may affect the lipid profile.

## 5. Conclusions

In recent years, growing scientific evidence has demonstrated specific biological activities of phytonutrients or phytochemicals, which can have a significant impact on the course of some diseases, particularly cardiovascular disease. This study was designed to reveal whether short-term consumption of bitter apricot seeds has any effect on lipid profile and LDL lipoprotein subfractions in adults with elevated total cholesterol.

The atherogenic lipoprotein profile might be present in about 6% of normolipidemic, young healthy individuals. Nonetheless, the main conclusion of this study was that after the six-week bitter apricot seeds consumption, the number of subjects with atherogenic fraction LDL_3–7_ decreased in both groups. The results of the present study support the hypothesis that daily consumption of bitter apricot seeds for 42 days positively modified the lipoprotein profile in the group with elevated total cholesterol and had no negative effect on lipid metabolism in the group of healthy probands.

Future studies to optimize the benefit of regular consumption of bitter apricot seeds and to determine the clinical relevance of metabolic change are justified as outlined in this study. Coupled with changes in traditional lipoprotein measurements, these improvements represent a favorable shift in the atherogenic phenotype and suggest that lifestyle interventions can improve early markers of atherosclerosis.

## Figures and Tables

**Table 1:** Content of nutrients and bioactive compounds of apricot seeds.

Organic Content (%)		Mineral Content (mg/100 g)		Content of Fatty Acids (%)		Content of Phenolic Compounds (mg/g)	
Dry matter	95.9	Ca	177.4	Oleic acid	64.5	Gallic acid	30.1
Protein	22.8	P	470.0	Linoleic acid	27.1	Ferulic acid	2.9
Ash	2.5	Mg	205.0	Palmitic acid	4.6	Cinnamic acid	0.9
Starch	2.3	Na	64.2	Stearic acid	1.2	Genistein	5.6
Total sugars	6.3	K	592.5	Palmitoleic acid	0.8	Rutin	11.3
Crude fiber	28.5	Fe	2.5	SFA	5.9		
Amygdalin	5.8	Zn	5.9	MUFA	65.3		
Oil	41.3	Mn	0.6	PUFA	27.1		

Abbreviations: SFA, saturated fatty acids; MUFA, monounsaturated fatty acids; PUFA, polyunsaturated fatty acids.

**Table 2:** Baseline characteristics of study participants.

Parameter	NTC (*n* = 21)		ETC (*n* = 13)	
	Women (*n* = 14)	Men (*n* = 7)	Women (*n* = 7)	Men (*n* = 6)
Age (y)	37.5 ± 9.13	33.14 ± 3.18	45.29 ± 14.10	41.50 ± 13.49
BW (kg)	60.54 ± 8.55	81.16 ± 11.41	66.21 ± 17.29	91.18 ± 17.17
VFA (cm^2^)	70.57 ± 20.60	60.76 ± 28.96	94.87 ± 37.21	106.85 ± 37.76
BMI (kg/m^2^)	22.00 ± 2.44	25.68 ± 3.16	24.70 ± 4.84	29.19 ± 3.98
T-C (mmol/L)	4.21 ± 0.57	4.31 ± 0.51	5.85 ± 0.64	5.80 ± 0.61
HDL-C (mmol/L)	1.59 ± 0.33	1.38 ± 0.29	1.81 ± 0.41	1.42 ± 0.23
LDL-C (mmol/L)	2.28 ± 0.49	2.42 ± 0.45	3.39 ± 0.38	3.84 ± 0.43
TG (mmol/L)	0.75 ± 0.31	1.15 ± 0.74	1.42 ± 0.75	1.21 ± 0.41
GLU (mmol/L)	5.06 ± 0.42	5.11 ± 0.12	5.01 ± 0.46	5.28 ± 0.79

Abbreviations: NTC, normal total cholesterol; ETC, elevated total cholesterol; BW, body weight; VFA, visceral fat area; BMI, body mass index; T-C, total cholesterol; HDL-C, high-density cholesterol; LDL-C, low-density cholesterol; TG, triglycerides; GLU, glucose. Data are expressed as mean ± standard deviation (SD).

**Table 3:** Anthropometric characteristics of the subjects before and after apricot seeds consumption.

Parameter	NTC (*n* = 21)			ETC (*n* = 13)		
	Day 0	Day 42	*p*-Value	Day 0	Day 42	*p*-Value
BW (kg)	67.41 ± 13.63	67.04 ± 13.46	0.0331	77.74 ± 20.98	78.34 ± 21.39	0.0243
BFM (kg)	16.27 ± 5.43	15.79 ± 5.29	0.0178	23.91 ± 8.67	24.45 ± 8.95	>0.05
BFM (%)	24.32 ± 7.02	23.70 ± 6.82	0.0234	30.70 ± 6.98	31.17 ± 6.53	>0.05
VFA (cm^2^)	67.30 ± 23.45	65.96 ± 22.94	>0.05	100.40 ± 36.40	103.56 ± 38.25	0.0344
BMI (kg/m^2^)	23.22 ± 3.16	23.10 ± 3.13	0.0463	26.77 ± 4.87	26.98 ± 5.02	0.0200
WHR	0.866 ± 0.04	0.861 ± 0.04	>0.05	0.944 ± 0.08	0.952 ± 0.07	>0.05
WC (cm)	82.0 ± 7.48	81.77 ± 7.32	>0.05	96.45 ± 15.77	96.90 ± 15.26	>0.05

Abbreviations: NTC, normal total cholesterol; ETC, elevated total cholesterol; BW, body weight; BFM, body fat mass; VFA, visceral fat area; BMI, body mass index. Data are expressed as mean ± standard deviation (SD). Paired *t*-test for normally distributed variables.

**Table 4:** Lipid profiles and fasting glucose of the subjects before and after apricot seeds consumption (mmol/L).

Parameter	NTC (*n* = 21)			ETC (*n* = 13)		
	Day 0	Day 42	*p*-Value	Day 0	Day 42	*p*-Value
T-C	4.25 ± 0.54	4.25 ± 0.65	>0.05	5.83 ± 0.60	5.48 ± 0.83	0.0200
LDL-C	2.33 ± 0.47	2.33 ± 0.56	>0.05	3.60 ± 0.45	3.22 ± 0.62	0.0045
HDL-C	1.52 ± 0.33	1.49 ± 0.31	>0.05	1.63 ± 0.39	1.67 ± 0.54	>0.05
TG	0.73 (0.56–0.80)	0.78 (0.63–0.92)	>0.05	1.31 (0.83–1.56)	1.23 (0.88–1.62)	>0.05
GLU	5.08 ± 0.35	5.24 ± 0.57	>0.05	5.13 ± 0.62	5.25 ± 0.61	>0.05

Abbreviations: NTC, normal total cholesterol; ETC, elevated total cholesterol; T-C, total cholesterol; HDL-C, high-density cholesterol; LDL-C, low-density cholesterol; TG, triglycerides; GLU, glucose. Data are expressed as mean ± standard deviation (SD) or median (interquartile range). Paired *t*-test for normally distributed variables and Wilcoxon Signed-Ranks Test for not normally distributed variables.

**Table 5:** Lipoprotein subfractions (mmol/L) and LDL particle size (nm) of the subjects before and after apricot seeds consumption.

Parameter	NTC (*n* = 21)			ETC (*n* = 13)		
	Day 0	Day 42	*p*-Value	Day 0	Day 42	*p*-Value
VLDL	0.81 ± 0.14	0.87 ± 0.20	>0.05	1.14 ± 0.19	1.06 ± 0.15	>0.05
IDL-A	0.57 ± 0.14	0.62 ± 0.14	>0.05	0.65 ± 0.26	0.76 ± 0.21	>0.05
IDL-B	0.31 ± 0.07	0.28 ± 0.08	0.0049	0.45 ± 0.16	0.46 ± 0.16	>0.05
IDL-C	0.43 ± 0.08	0.46 ± 0.09	>0.05	0.62 ± 0.06	0.64 ± 0.18	>0.05
LDL_1_	0.77 ± 0.18	0.72 ± 0.17	>0.05	1.16 ± 0.22	1.00 ± 0.21	0.0035
LDL_2_	0.15 (0.07–0.21)	0.08 (0.05–0.15)	0.0393	0.52 (0.30–0.63)	0.18 (0.13–0.38)	0.0002
LDL_3–7_	0 (0–0)	0 (0–0)	>0.05	0 (0–0.05)	0 (0–0)	0.0431
HDL	1.15 ± 0.20	1.12 ± 0.22	>0.05	1.33 ± 0.26	1.26 ± 0.30	>0.05
Total LDL	2.29 ± 0.38	2.25 ± 0.44	>0.05	3.39 ± 0.43	3.09 ± 0.54	0.0019
Mean LDL particle size	27.39 ± 0.27	27.48 ± 0.24	0.0326	27.24 ± 0.21	27.43 ± 0.14	0.0015

Abbreviations: NTC, normal total cholesterol; ETC, elevated total cholesterol; VLDL, very low-density lipoproteins; IDL, intermediate-density lipoproteins; LDL, low-density lipoproteins; HDL, high-density lipoproteins. Data are expressed as mean ± standard deviation (SD) or median (interquartile range). Paired *t*-test for normally distributed variables and Wilcoxon Signed-Ranks Test for not normally distributed variables.

**Table 6:** High-sensitivity CRP, CK, and liver enzymes of the subjects before and after apricot seeds consumption.

Parameter	NTC (*n* = 21)			ETC (*n* = 13)		
	Day 0	Day 42	*p*-Value	Day 0	Day 42	*p*-Value
hs-CRP (mg/L)	0.26 (0.14–0.61)	0.50 (0.21–0.98)	>0.05	1.46 (0.62–3.19)	1.28 (0.62–3.24)	>0.05
CK (mg/L)	1.93 ± 1.03	2.09 ± 1.19	>0.05	1.76 ± 0.96	1.94 ± 0.89	>0.05
ALP (µkat/L)	0.80 ± 0.31	0.82 ± 0.29	>0.05	0.97 ± 0.16	1.01 ± 0.20	>0.05
AST (µkat/L)	0.36 ± 0.11	0.31 ± 0.08	0.0075	0.39 ± 0.09	0.38 ± 0.11	>0.05
ALT (µkat/L)	0.35 ± 0.16	0.33 ± 0.1	>0.05	0.45 ± 0.29	0.47 ± 0.21	>0.05
GGT (µkat/L)	0.27 ± 0.20	0.35 ± 0.14	0.0370	0.45 ± 0.30	0.46 ± 0.27	>0.05

Abbreviations: NTC, normal total cholesterol; ETC, elevated total cholesterol; hs-CRP, high-sensitivity C-reactive protein; CK, creatine kinase; ALP, alkaline phosphatase; AST, aspartate aminotransferase; ALT, alanine aminotransferase; GGT, gamma glutamyl transferase. Data are expressed as mean ± standard deviation (SD) or median (interquartile range). Paired *t*-test for normally distributed variables and Wilcoxon Signed-Ranks Test for not normally distributed variables.

**Table 7:** Changes of monitored parameters after nutrition intervention adjusted for body mass index (BMI) and sex.

Parameter	NTC (*n* = 21)			ETC (*n* = 13)		
	Difference ^a^	*p*-Value ^b^	*p*-Value ^c^	Difference ^a^	*p*-Value ^b^	*p*-Value ^c^
T-C (mmol/L)	0.00 ± 0.44	0.0290	>0.05	0.35 ± 0.47	>0.05	>0.05
LDL-C (mmol/L)	−0.01 ± 0.40	0.013	>0.05	0.38 ± 0.39	>0.05	>0.05
HDL-C (mmol/L)	0.02 ± 0.13	>0.05	>0.05	−0.04 ± 0.19	0.014	>0.05
TG (mmol/L)	−0.05 ± 0.22	>0.05	>0.05	0.04 ± 0.42	>0.05	>0.05
VLDL (mmol/L)	−0.06 ± 0.18	0.0087	0.0100	0.08 ± 0.19	>0.05	>0.05
IDL-C (mmol/L)	−0.04 ± 0.11	0.0078	>0.05	−0.03 ± 0.16	>0.05	>0.05
IDL-B (mmol/L)	0.03 ± 0.04	>0.05	>0.05	−0.01 ± 0.08	>0.05	>0.05
IDL-A (mmol/L)	−0.05 ± 0.13	0.0355	>0.05	−0.11 ± 0.22	>0.05	>0.05
LDL_1_ (mmol/L)	0.05 ± 0.16	>0.05	>0.05	0.16 ± 0.16	>0.05	>0.05
LDL_2_ (mmol/L)	0.06 ± 0.13	>0.05	>0.05	0.28 ± 0.20	>0.05	>0.05
LDL_3–7_ (mmol/L)	0.00 ± 0.05	>0.05	>0.05	0.02 ± 0.03	>0.05	>0.05
LDL particle size (nm)	−0.86 ± 1.71	>0.05	>0.05	−1.92 ± 1.71	>0.05	>0.05
GLU (mmol/L)	−0.17 ± 0.50	0.0243	>0.05	−0.12 ± 0.43	>0.05	>0.05
AST (µkat/L)	0.06 ± 0.09	>0.05	>0.05	0.01 ± 0.08	>0.05	>0.05
ALT (µkat/L)	0.02 ± 0.10	>0.05	>0.05	−0.02 ± 0.17	>0.05	>0.05
ALP (µkat/L)	−0.03 ± 0.09	>0.05	>0.05	−0.05 ± 0.08	>0.05	>0.05
GGT (µkat/L))	−0.08 ± 0.16	>0.05	0.0427	−0.01 ± 0.08	>0.05	>0.05
CK (mg/L)	−0.16 ± 0.42	>0.05	>0.05	−0.18 ± 0.79	>0.05	>0.05
*h*s-CRP (mg/L)	−0.04 ± 1.01	>0.05	>0.05	0.07 ± 0.76	>0.05	>0.05

Abbreviations: NTC, normal total cholesterol; ETC, elevated total cholesterol; BMI, body mass index; T-C, total cholesterol; HDL-C, high-density cholesterol; LDL-C, low-density cholesterol; TG, triglycerides; VLDL, very low-density lipoproteins; IDL, intermediate-density lipoproteins; GLU, glucose; AST, aspartate aminotransferase; ALT, alanine aminotransferase; ALP, alkaline phosphatase; GGT, gamma glutamyl transferase; CK, creatine kinase; hs-CRP, high-sensitivity C-reactive protein. Data are expressed as mean ± standard deviation (SD); ^a^ difference between pre- and post- intervention. All *p*-values ^b,c^ are obtained from the ANCOVA analysis with adjustment for baseline values (covariates ^b^ sex, ^c^ BMI).

## Data Availability

All datasets related to the results of this study are available from the primary author on request.
